# Renewals

**Published:** 1885-11-20

**Authors:** 


					﻿EDITORIALS AND MISCELLANEOUS.
Renewals.—We remind our friends that the time for renewing their
subscriptions is at hand. We take for granted that all will renew ; not only so,
but that each subscriber will send us a new subscriber. To any subscriber who
will send us $3, on renewing nis subscription, we will send two copies of the
Record one year—one copy for himself and one foi his friend.
				

